# Reply to Sánchez-Pacheco et al., Chookajorn, and Mavian et al.: Explaining phylogenetic network analysis of SARS-CoV-2 genomes

**DOI:** 10.1073/pnas.2007433117

**Published:** 2020-05-21

**Authors:** Peter Forster, Lucy Forster, Colin Renfrew, Michael Forster

**Affiliations:** ^a^Institute of Forensic Genetics, 48161 Münster, Germany;; ^b^McDonald Institute for Archaeological Research, University of Cambridge, Cambridge CB2 3ER, United Kingdom;; ^c^Fluxus Technology Limited, Colchester CO3 0NU, United Kingdom;; ^d^Lakeside Healthcare Group at Cedar House Surgery, St Neots PE19 1BQ, United Kingdom;; ^e^Institute of Clinical Molecular Biology, Christian-Albrecht-University of Kiel, 24105 Kiel, Germany

We calculated a phylogenetic analysis network of the 160 severe acute respiratory syndrome coronavirus 2 (SARS-CoV-2) complete genomes submitted to the international Global Initiative on Sharing Avian Influenza Data (GISAID) database by early March 2020, to produce a snapshot of the beginning of the SARS-CoV-2 epidemic (1). Phylogenetic network analysis of human mitochondrial DNA and human Y chromosomes has been successful in revealing the prehistoric spread of *Homo sapiens* across the planet (2, 3), and we reasoned it would equally be a useful tool for reconstructing the mutational evolution of coronavirus genomes. Combining median-joining and Steiner network algorithms, we obtained a network visualizing the 288 most parsimonious trees simultaneously. Included in the dataset was the closest known nonhuman coronavirus, a bat coronavirus with more than 96% sequence similarity to the human virus. This bat coronavirus rooted the network in a cluster we have labeled “A,” from which a prominent cluster “B” and in turn a prominent cluster “C” derive. In these 160 genomes representing the initial phase of the epidemic, within China the ancestral A types are more common outside Wuhan rather than within Wuhan. We observe that documented transmission paths closely follow the order of mutations that is inferred by the network.

## Reply to Sánchez-Pacheco et al.

The letter by Sánchez-Pacheco et al. (4) consists of a number of unsubstantiated statements with regard to coronavirus evolutionary analysis, and of methodological misunderstandings, as follows.

Sánchez-Pacheco et al. (4) opine that a network does not reflect the important biological features thought to underlie viral evolution, such as recombination and horizontal gene transfer, making median-joining networks inappropriate in this setting. However, neither Sánchez-Pacheco et al. nor we in our PNAS paper (1) claim that recombination in the human coronavirus data has happened. It is therefore not clear why Sánchez-Pacheco et al. raise this point. In fact, if recombination had happened frequently, then the data would be expected to contain extensive character conflicts, which in turn would generate a network with extensive reticulations and hypercubes, making the network method a useful diagnostic tool for such events.

Sánchez-Pacheco et al. (4) consider that the cycles present in a median-joining network provide no information about the evolutionary history of the sequences because of the absence of direction. However, we contend that the inherent advantage of a network over any single tree is to display unresolved data conflicts as cycles (reticulations), allowing the visualization in this case of 288 most parsimonious coronavirus trees at one glance (figure 1 in ref. 1).

Sánchez-Pacheco et al. (4) state that phylogenies do not directly trace transmission history. This is clearly true as a general point but evidently not true with respect to the rapidly mutating coronavirus genome. The virus mutates faster than one mutation per month, which is a short time relative to the serial interval of 4 to 8 d in Sars-CoV-2 infection chains (5). It therefore makes sense that the network mutations closely reflect infection pathways, as we have shown in our published casework.

Sánchez-Pacheco et al. (4) report that the implication that median-joining networks reflect phylogenetic signal in the traditional sense has previously been challenged in one study. We refer to the phylogenetic signal being clearly demonstrated in our published documented case studies as well as in the geographic specificities of each cluster A, B, and C.

Sánchez-Pacheco et al. (4) have the impression that our “outgroup does not root at A, but rather A itself is derived from one of two possible ancestral viruses with this rooting.” However, this is a misreading of figure 1 in ref. 1. The label “A” refers to the cluster as defined in the text of our article, not to one particular node.

Sánchez-Pacheco et al. (4) erroneously believe that the median-joining software option used for our rooting merely links the “outgroup” sequence (the bat coronavirus) to the most similar sequence of the already-produced “ingroup” network.” In fact, however, we did not use this “post-network rooting” option and make no mention of it in our paper. We ran the bat sequence as part of the human dataset.

## Reply to Chookajorn

The letter by Chookajorn (6) praises our network method for its successes in human evolutionary studies. Chookajorn then goes on to endorse our coronavirus network insofar as our clusters are reproducible in an independent maximum likelihood approach (7). Chookajorn in his concluding paragraph then states his concerns that sensational scientific results can influence decision making and he states that “any potential misinformation must be promptly addressed.” Presumably he is referring to the use of our article by certain media, who have tended, in several recent interviews and newspaper articles, to interpret our article as “evidence” for an American origin of the coronavirus. We have spoken out against this interpretation of our results. So, we have no disagreement with Chookajorn on this point.

## Reply to Mavian et al.

Mavian et al. (8) support our observation of geographic clustering but make multiple mistakes, starting with the rooting of the coronavirus phylogeny.

They declare that the sequence identity between SARS-CoV-2 and the bat virus is only 96.2%, implying that these viral genomes (which are nearly 30,000 nucleotides long) differ by more than 1,000 mutations. In their view, such a distant outgroup is unlikely to provide a reliable root for the network. We argue, on the contrary, that the bat virus is surprisingly conclusive, as shown by its stable rooting in cluster A despite incrementally increasing the epsilon “fuzziness” setting in the median-joining network algorithm as described in PNAS (1). Where Mavian et al. (8) have gone wrong is to look no further than the 3.8% difference between bat and human coronavirus differences. However, if they had considered that the bat virus genome is 30,000 nucleotides long, and then had looked at the 1,200 nucleotide differences between bat and human virus, they would have seen that only 19 nucleotides are shared polymorphisms between the bat coronavirus and the consensus of the 160 human coronavirus genomes, encompassing maximum parsimony trees of 212 mutations. On this basis, the bat coronavirus is an excellent outgroup for rooting the network. Furthermore, since publication we have confirmed the bat coronavirus rooting with two strains of the more distant pangolin coronavirus.

Mavian et al. (8) are puzzled why the branch to the bat virus, in figure 1 of ref. 1, is only 16 or 17 mutations in length. The answer is that we had stripped all private polymorphisms from the bat coronavirus before running it as an outgroup.

Mavian et al. (8) refer to SI Appendix, figure S4 in ref. 1 and contend that the network seems to be misrooted because a virus from Wuhan from week 0 (December 24, 2019) is portrayed as a descendant of a clade of viruses collected in weeks 1 through 9. However, this assumption, that the oldest sampled isolate in a cohort reflects the ancestral type, is a misconception by Mavian et al. The first isolates collected from patients starting on December 24 do not reflect the root type of the outbreak, which started weeks or months earlier. The purpose of SI Appendix, figure S4 in ref. 1 is to demonstrate the futility of using the sampling date of each patient to reconstruct the virus phylogeny, at least in this phase of the outbreak.

Mavian et al. (8) then misread our article several times, confusing the mutations and amino acid changes distinguishing between A, B, and C.

Mavian et al. (8) reproach us by stating that SARS-CoV-2 sequences showing some geographical clustering cannot be used as a proof of biological differences unless backed by solid experimental data (6). Here again, Mavian et al. have misread our article: They reiterate one aspect of our article, but phrase it as a reproach. The correct reading of our article is that we encourage experimental researchers to consider one of the possible explanations, namely a biological effect of the mutations.

Mavian et al. (8) state that our findings are based on a nonrepresentative dataset of 160 genomes, with no significant correlation between prevalence of confirmed cases and number of sequenced strains per country. We respond that our data are based on the first 160 high-quality genomes collected, sequenced, and uploaded to the international GISAID database, in order to shed light on the early development of the coronavirus. The early outbreak was centered on China, and naturally in this early dataset China is well represented. It is not clear which alternative sampling strategies Mavian et al. have in mind or what their alternative sampling strategy would achieve.

Finally, Mavian et al. (8) caution that no firm conclusion should be drawn on disease transmission routes without evaluating the probability of alternative dissemination routes. In general we would agree with this point, but here we are dealing with the very first detected infections in several countries in January and February 2020. Thus, there are no realistic alternatives to be evaluated. The first Mexican case had traveled to Italy, and the network shows his viral type descended from an Italian viral type. The early Canadian patient had traveled to Wuhan and Guangdong, and the network shows his type to be descended from a Guangdong node. The first Brazilian patient had traveled to Italy, and his type is descended from an Italian type. This clear picture is initially surprising but makes sense from a mutational point of view: The virus mutates faster than one mutation per month, which is a short time relative to the serial interval of 4 to 8 d in Sars-CoV-2 infection chains (5). It therefore makes sense that the network mutations closely reflect infection pathways.
